# Quantitative myocardial perfusion in coronary artery disease: A perfusion mapping study

**DOI:** 10.1002/jmri.26668

**Published:** 2019-01-25

**Authors:** Kristopher D. Knott, Claudia Camaioni, Anantharaman Ramasamy, Joao A. Augusto, Anish N. Bhuva, Hui Xue, Charlotte Manisty, Rebecca K. Hughes, Louise A.E. Brown, Rajiv Amersey, Christos Bourantas, Peter Kellman, Sven Plein, James C Moon

**Affiliations:** ^1^ University College London Institute of Cardiovascular Science London UK; ^2^ Barts Heart Centre St Bartholomew's Hospital London UK; ^3^ National Heart, Lung, and Blood Institute National Institutes of Health, DHHS Bethesda Maryland USA; ^4^ Department of Biomedical Imaging Science, Leeds Institute of Cardiovascular and Metabolic Medicine University of Leeds Leeds UK

**Keywords:** myocardial perfusion, perfusion mapping, coronary artery disease, inline perfusion quantification, cardiovascular magnetic resonance

## Abstract

**Background:**

Cardiac MR stress perfusion remains a qualitative technique in clinical practice due to technical and postprocessing challenges. However, automated inline perfusion mapping now permits myocardial blood flow (MBF, ml/g/min) quantification on‐the‐fly without user input.

**Purpose:**

To investigate the diagnostic performance of this novel technique in detecting occlusive coronary artery disease (CAD) in patients scheduled to undergo coronary angiography.

**Study Type:**

Prospective, observational.

**Subjects:**

Fifty patients with suspected CAD and 24 healthy volunteers.

**Field Strength:**

1.5T.

**Sequence:**

"Dual" sequence multislice 2D saturation recovery.

**Assessment:**

All patients underwent cardiac MR with perfusion mapping and invasive coronary angiography; the healthy volunteers had MR with perfusion mapping alone.

**Statistical Tests:**

Comparison between numerical variables was performed using an independent *t*‐test. Receiver operator characteristic (ROC) curves were generated for transmyocardial, endocardial stress MBF, and myocardial perfusion reserve (MPR, the stress:rest MBF ratio) to diagnose severe (>70%) stenoses as measured by 3D quantitative coronary angiography (QCA). ROC curves were compared by the method of DeLong et al.

**Results:**

Compared with volunteers, patients had lower stress MBF and MPR even in vessels with <50% stenosis (2.00 vs. 3.08 ml/g/min, respectively). As stenosis severity increased (<50%, 50–70%, >70%), MBF and MPR decreased. To diagnose occlusive (>70%) CAD, endocardial and transmyocardial stress MBF were superior to MPR (area under the curve 0.92 [95% CI 0.86–0.97] vs. 0.90 [95% CI 0.84–0.95] and 0.80 [95% CI 0.72–0.87], respectively). An endocardial threshold of 1.31 ml/g/min provided a per‐coronary artery sensitivity, specificity, positive predictive value (PPV), and negative predictive value (NPV) of 90%, 82%, 50%, and 98%, with a per‐patient diagnostic performance of 100%, 66%, 57%, and 100%, respectively.

**Data Conclusion:**

Perfusion mapping can diagnose occlusive CAD with high accuracy and, in particular, high sensitivity and NPV make it a potential "rule‐out" test.

**Level of Evidence:** 1

**Technical Efficacy Stage:** 2 J. Magn. Reson. Imaging 2019;50:756–762.

Coronary artery disease (CAD) remains a major global cause of morbidity and mortality.^1^ Functional noninvasive testing permits CAD detection by identifying areas of impaired myocardial perfusion at stress that may benefit from revascularization.^2^, ^3^, ^4^ This can help to reduce the invasive angiography rates and better targets invasive strategies. However, for effective care noninvasive testing needs to be accurate.^5^

Myocardial perfusion cardiac magnetic resonance imaging (MRI) is a well‐validated functional ischemia test and is widely used.^6^, ^7^ Images are acquired during the first myocardial passage of a gadolinium‐based contrast agent during vasodilator stress and often repeated at rest. Areas of hypoperfusion at stress indicate ischemia. However, in clinical practice interpretation of myocardial perfusion MRI is mostly qualitative, relying on an experienced operator to identify true perfusion defects. Quantifying myocardial blood flow (MBF) has the potential to be less operator‐dependent^8^ and to better detect balanced ischemia in three‐vessel disease.^9^ Fully quantitative perfusion using positron emission tomography (PET) has shown additional prognostic benefits in CAD and cardiomyopathy.^10^, ^11^ However, PET uses ionizing radiation, is more expensive, and there is often limited availability in many regions.

Developing quantitative perfusion MRI for clinical use has been challenging. Dual bolus or dual sequence approaches^12^, ^13^, ^14^ are needed for quantification in order to obtain the arterial input function. Subsequently, there is laborious operator input, often requiring the contouring of up to 300 images per patient. Therefore, the technique has not transitioned to clinical care. Offline techniques are improving, but operator input and image porting to custom tools remains necessary.^15^ Recently, these limitations have been effectively overcome with inline "Perfusion Mapping."^16^ This method uses a dual sequence approach, with respiratory motion correction and signal nonlinearity correction. Exploiting modern computing hardware, there is automatic identification of the left ventricle (LV) blood pool and generation of the arterial input function. A sophisticated blood tissue exchange model delivers pixel‐by‐pixel blood flow quantification solved using partial differential equations.^17^ Within ~90 seconds, perfusion maps are outputted on the scanner, where each voxel color encodes myocardial blood flow (in ml/g/min, Fig. 1). The technique has been validated against PET, showing good correlation.^18^, ^19^

**Figure 1:**
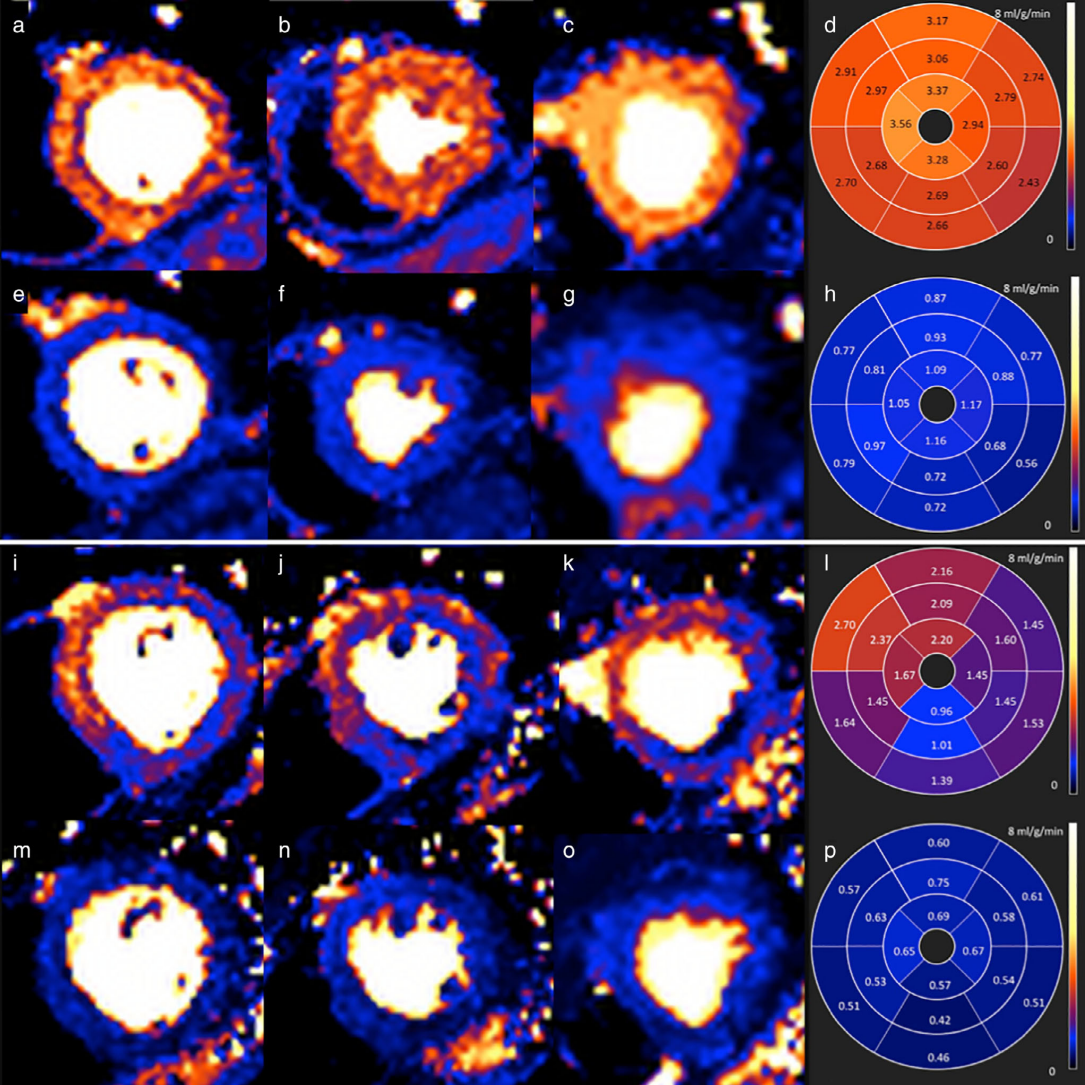
Perfusion maps in health and disease. Stress (a–c) and rest (e–g) perfusion maps for a 64‐year‐old healthy volunteer and a patient with 80% stenosis of the LCx and occlusion of the RCA (i–k,m–o). The polar maps (l,p) indicate that the patient's stress MBF is lowest in the RCA territory (0.96 ml/g/min) and 2.09–2.70 ml/g/min in remote myocardium. The volunteer's stress MBF is 2.43–3.17 ml/g/min and rest MBF 0.42–0.79 ml/g/min.

We investigated the diagnostic performance of perfusion mapping in patients suspected of having coronary disease.

## Materials and Methods

The study was approved by the National Health Service Research Ethics Committee (NHS REC) and Health Research Authority (HRA) and conducted in accordance with the Declaration of Helsinki. All subjects provided written, informed consent. Fifty patients with chest pain and scheduled for invasive coronary angiography were prospectively recruited. All patients underwent MRI with automated inline perfusion mapping prior to invasive angiography. The exclusion criteria were previous coronary artery bypass grafting, chronic kidney disease stage IV and V (estimated glomerular filtration rate [eGFR] <30 mmol/l), overt cardiomyopathy (hypertrophic, arrhythmogenic, dilated, amyloid), contraindication to MRI, or contraindication to adenosine. A healthy volunteer cohort of 24 individuals with no cardiac symptoms, medications, or comorbidities was also recruited prospectively.

### *MRI Protocol*

MRI scans were performed at 1.5 T (Aera, Siemens Healthineers, Erlangen, Germany) using a standard clinical protocol.^20^ The protocol consisted of cine imaging, stress and rest perfusion, and late gadolinium enhancement (LGE). For the perfusion imaging, adenosine was infused for 4 minutes at a dose of 140 μg/kg/min (increased to 175 μg/kg/min for a further 2 min if less than 10% heart rate increase or no symptoms). At peak stress a gadolinium‐based contrast agent (gadoterate meglumine, Dotarem, Guerbet, Paris, France) was injected into a peripheral vein at 4 ml/s at a dose of 0.05 mmol/kg and 60 dynamic images were acquired for three LV short‐axis slices. Rest perfusion images were acquired after an interval of 6–10 minutes. Perfusion mapping was performed as previously described^16^ and implemented on the scanner using the Gadgetron streaming software image reconstruction framework.^21^

**Figure 2:**
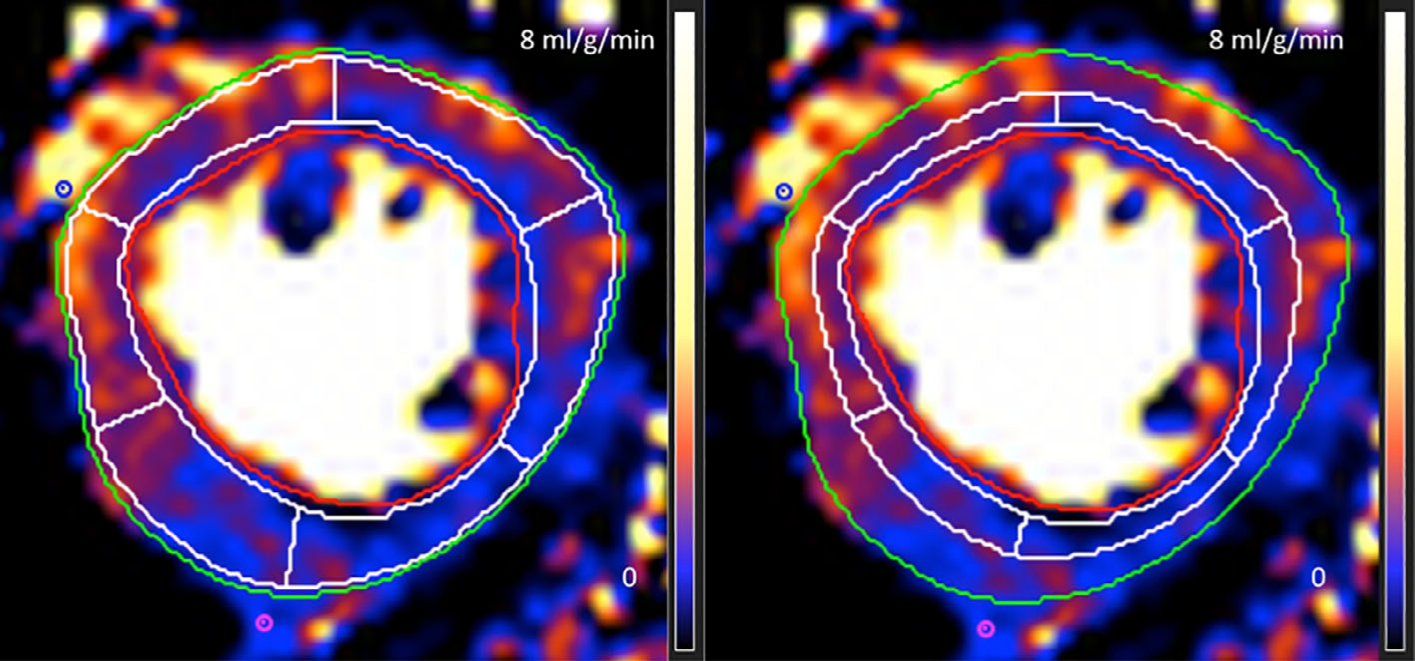
Perfusion map analysis. An example of perfusion map analysis for a single mid‐LV slice. Endocardial and epicardial borders were manually traced, RV insertion points identified, and the LV segmented. Left panel: The borders are offset by 10% to minimize partial volume effects at the blood‐myocardial and myocardial‐pericardial borders. Right panel: The epicardial border is offset by 50% to measure endocardial flow.

The perfusion maps were analyzed using commercially available software (CVI42, Circle Cardiovascular Imaging, Calgary, Canada). This consisted of simply contouring the endo‐ and epicardial on three slices and defining the right ventricular (RV) insertion points. The software created a border 10% offset and drew the 16 segment American Heart Association (AHA) model.^22^ By placing the epicardial offset to 50%, an endocardial and epicardial MBF was also obtained (Fig. 2). The mean MBF of the two myocardial segments with the lowest flow in each coronary territory was calculated to ensure that there was no bias against distal coronary stenosis.^23^ The MBF was calculated in this way for each coronary territory at stress and rest and the ratio of these gave the myocardial perfusion reserve (MPR). Segments with subendocardial and transmural LGE were excluded from the analysis but wall motion abnormalities were not considered.

Finally, the data were analyzed on a per‐patient basis and the scan was declared abnormal if there was a reduced stress MBF/MPR in any coronary territory.

### *Invasive Angiography*

Invasive angiography was performed according to the standard clinical protocols. The major epicardial vessels and large side branches (intermediate, large diagonal, and large obtuse marginal) with visual luminal stenosis >30% were reconstructed based on the angiographic data using 3D quantitative coronary angiography (3D QCA) methodology.^24^ This was performed using well‐validated software (QAngio XA 3D RE, Medis Specials, Leiden, The Netherlands). Two end‐diastolic angiographic projections (>25° apart) with no overlapping or foreshortening of the segment of interest were selected for analysis to allow accurate delineation of the lumen silhouette. The lumen centerline and borders were automatically detected in both projections using an established edge detection algorithm. Manual adjustments were made when needed by an experienced interventional cardiologist blinded to MRI results. For each studied segment the reference lumen area, minimum lumen area and lesion length were estimated and the diameter stenosis (DS) was calculated. The studied lesions were grouped into three DS groups: DS <50% (mild), DS 50–70% (moderate), and DS >70% (severe).

### *Statistical Analysis*

Statistical analysis was performed in SPSS (IBM SPSS statistics, v. 25.0, Armonk, NY). Numerical variables are presented as mean ± standard deviation and categorical variables as absolute values and percentages. Comparison between numerical variables was performed using an independent *t*‐test while the chi‐square test was used for categorical variables. *P* < 0.05 was considered statistically significant. Receiver operator characteristic (ROC) curves were calculated to determine the accuracy of the perfusion maps in detecting a DS >70% in a coronary artery and determine the optimal cutoffs for diagnosis of severe stenosis (>70%). Subsequently, the sensitivity, specificity, positive predictive value (PPV), and negative predictive value (NPV) in diagnosing a patient with CAD on the basis of quantitative perfusion was calculated. The area under the curve (AUC) of ROC curves were compared using the method of DeLong et al.^25^

## Results

Patient and volunteer characteristics are displayed in Table 1. Patients were older (58.2 vs. 37.3 years, *P* < 0.001), a higher proportion were male (87% vs. 50%, *P* = 0.003), and had more comorbidities compared with the volunteers.

**Table 1:** Patient and Volunteer Characteristics

	Patients (*n* = 50)	Volunteers (*n* = 24)	*P* value
Age (years)	58.2	37.3	<0.001
Gender (% male)	86	50	0.003
Height (cm)	172	173	0.556
Weight (kg)	83	77	0.083
BSA	1.99	1.92	0.181
LVEDV (ml)	145	153	0.389
LV mass (g)	116	103	0.090
EF (%)	66	66	0.924
Diabetes (%)	16	0	0.004
Hypertension (%)	58	0	<0.001
Hypercholesterolemia (%)	68	0	<0.001
Smoker (%)	46	0	<0.001
AF (%)	6	0	0.226

Patients were significantly older, a greater proportion were male, and they had more comorbidities than the volunteers. Body surface area (BSA), left ventricular (LV), EDV (end diastolic volume), ejection fraction (EF), atrial fibrillation (AF).

Perfusion mapping analysis was feasible in all 150 coronary territories. Conversely, 3D QCA analysis was not performed in 28 vessels, as in these cases it was not possible to obtain two angiographic views 25° apart, with no foreshortening or overlapping of the segment of interest. Therefore, 122 vessels and their corresponding myocardial territories were included in the final analysis. Stenosis by 3D QCA was <50% in 81 vessels, 50–70% in 20 vessels, and > 70% in 21 vessels. Eighteen patients (40%) had at least one vessel with a severe stenosis (>70%). The healthy volunteer comparator cohort only had perfusion MRI and all 72 coronary territories could be analyzed by perfusion mapping.

**Figure 3:**
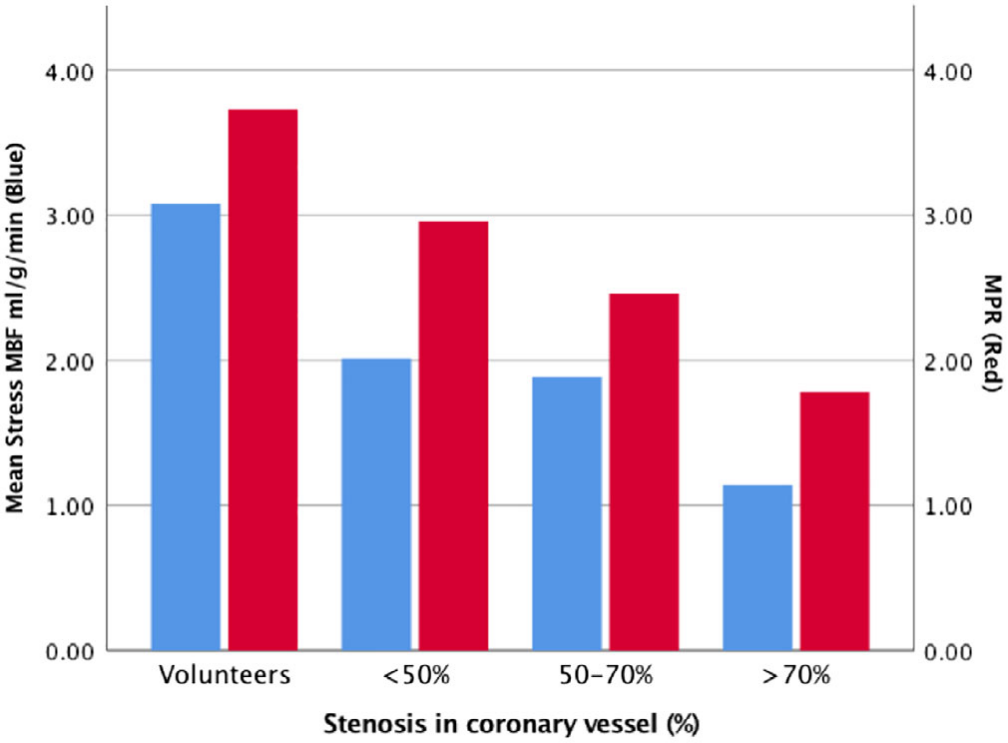
Myocardial blood flow and perfusion reserve in volunteers and patients. Stress MBF is lower for patients than volunteers (*P* < 0.001), even in territories with <50% stenosis. Stress MBF is significantly lower in vessels with >70% stenosis than <50% (*P* < 0.001) and 50–70% stenosis (*P* < 0.001). MPR is lower in patients than volunteers (*P* = 0.009). MPR is lower in vessels with >70% stenosis than <50% (*P* < 0.001) and 50–70% stenosis (*P* = 0.03).

Healthy volunteers had higher transmyocardial stress MBF and MPR than patients, even in segments supplied by vessels with <50% stenosis. Mean stress MBF was 3.07 ml/g/min in healthy volunteers and 2.00 ml/g/min in the myocardium of patients with DS <50%. As the DS increased, the MBF and MPR decreased (Fig. 3). Rest MBF was not significantly different between volunteers (0.86 ml/g/min) and remote myocardium (0.80 ml/g/min) or ischemic myocardium (0.77 ml/g/min), (*P* = 0.32 and *P* = 0.24, respectively).

**Figure 4:**
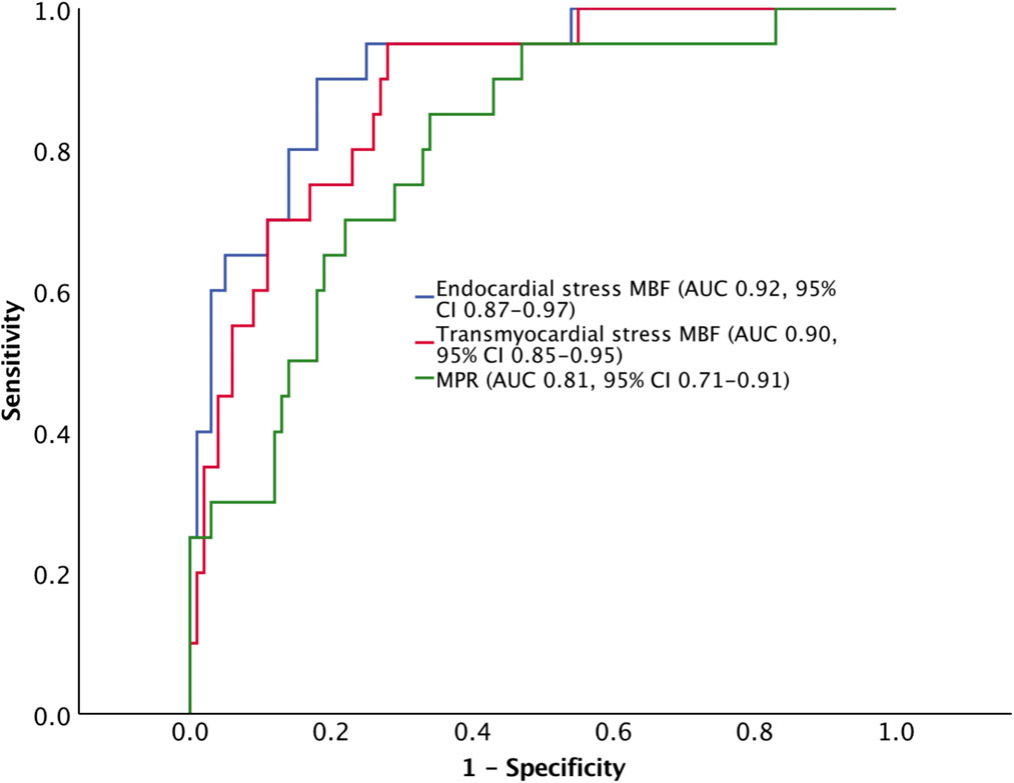
ROC curves plotting the sensitivity against 1‐ specificity for transmyocardial stress MBF, subendocardial MBF, and MPR in diagnosing a coronary stenosis >70%. Endocardial stress and transmyocardial stress are superior to MPR (*P* = 0.01 and *P* = 0.04, respectively).

ROC curves were calculated to determine the diagnostic accuracy of endocardial stress, transmyocardial stress MBF, and MPR for >70% stenoses per vessel (Fig. 4). Segments with LGE were excluded (eight myocardial territories). The accuracy of endocardial stress MBF (AUC 0.92, 95% confidence interval [CI] 0.87–0.97) was not statistically significantly different (*P* = 0.051) from transmyocardial stress MBF (AUC 0.90, 95% CI 0.84–0.95). Endocardial and transmyocardial stress was more accurate than MPR (AUC 0.81, 95% CI 0.71–0.91, *P* = 0.01 and *P* = 0.04, respectively).

An optimal diagnostic threshold for each parameter was determined from the ROC curves. The optimal threshold for endocardial stress on a per‐vessel basis was 1.31 ml/g/min, providing sensitivity, specificity, PPV, and NPV of 90%, 85%, 55%, and 98%, respectively; for transmyocardial MBF it was 1.50 ml/g/min, providing 90%, 78%, 47%, and 97%, respectively.

A per‐patient‐based analysis (rather than a single coronary vessel basis) was performed on the 45 patients where there was full QCA or where vessels that could not undergo QCA were obviously visually normal. The optimal cutoff point to declare the study abnormal for endocardial stress MBF was <1.31 ml/g/min with per‐patient sensitivity, specificity, PPV, and NPV of 100%, 74%, 73%, and 100%, respectively. Using the optimal cutoff point MBF of <1.5 ml/g/min in any coronary territory for transmyocardial stress, the sensitivity, specificity, PPV, and NPV was 100%, 70%%, 70%, and 100%, respectively.

## Discussion

In this study we demonstrated that stress perfusion MRI with automated inline perfusion mapping is feasible and accurate for the detection of significant CAD. Stress endocardial and transmyocardial MBF were the most accurate parameters, while MPR was less accurate. On both a coronary territory and whole patient basis, perfusion mapping was highly sensitive, and adequately specific with a high NPV. In our study, when MBF in every myocardial territory was >1.5 ml/g/min there was no occlusive CAD and this can be immediately visualized on the color perfusion maps. The concordance with angiography is important because it provides confidence in the technique for transition to clinical practice and complements the MRI validation studies against PET.^18^, ^19^

One of the major advantages of fully quantitative perfusion is that it removes the subjectivity in the interpretation of perfusion MRI. Perfusion mapping is a further step forward in that it is does not require the laborious postprocessing associated with other dual sequence techniques. As it uses a single contrast bolus approach, it is also suitable for the clinical workflow where dual bolus techniques can be cumbersome. These factors mean that perfusion mapping is well placed to be introduced into clinical MR. With this in mind, a key finding in our study is the high diagnostic accuracy of perfusion mapping. Using a completely automated approach, perfusion mapping achieves similar accuracies for the diagnosis of CAD to that achieved by expert visual reads in recent landmark studies such as CE‐MARC.^7^ This is an advance on what has been done previously.

Furthermore, the output of the color perfusion maps is another advantage of the approach. In a similar way that parametric mapping has improved tissue characterization,^26^ with an appropriate color look‐up table, perfusion mapping gives an easy, instantaneous visual representation of perfusion. As there was a very high NPV observed in this study, with the use of an appropriate color look‐up table, an observer can rapidly appreciate a normal scan. The high NPV of a normal quantitative perfusion study is also reflected in the PET literature.^27^

A degree of variation in absolute MBF and MPR is to be expected in any given population and is seen in all noninvasive tests, with heterogeneity introduced by variations in study protocols, sequences, and contrast or tracers. However, despite this, the MBF values we found in our study (both optimal cutoff values and our healthy volunteer results) are similar to those observed in the PET literature.^27^

It has been argued by some that that the MPR (the ratio of stress MBF to rest MBF) would be the best myocardial parameter of occlusive epicardial CAD. However, in our study (and others^27^), this was not the case. We found that it is the peak MBF that correlated best. MPR seems to fall short because the denominator of MPR, rest flow, is independently autonomically regulated and influenced by factors such as gender, resting heart rate, contractility, and wall stress that are not related to peak flow.

Areas of ischemia from an epicardial stenosis >70% have lower measurable MBF, which is readily visualized on the perfusion maps. However, perfusion is not absolutely specific to epicardial coronary flow. This may explain why the specificity and PPV were lower than the sensitivity and NPV in our study. "False positives" when using a fixed threshold approach may be a result of microvascular disease,^28^ submaximal vasodilatation, or a combination of these factors. Epicardial coronary disease and microvascular disease are not mutually exclusive and PET studies have shown that myocardial perfusion falls with an increasing coronary calcium score.^29^ Therefore, it is not surprising that there were patients in our cohort who had a significant coronary stenosis in a single vessel but additionally had impaired perfusion in other areas of the myocardium, perhaps due to microvascular disease. A result such as this would have resulted in a false positive on per‐vessel analysis but a true positive on per‐patient analysis.

There are limitations to our study, with our relatively small sample size and the inherent limitations in comparing an anatomical test (3D QCA) with a functional test (perfusion MRI). "False positive" perfusion maps may have actually been "false negative" 3D QCA analysis and vice versa; thus, artificially lowering our diagnostic accuracy. QCA is not a perfect truth standard but has been commonly used as an endpoint in major perfusion MRI trials.^4^, ^7^ This may also explain why there was only a trend towards significance in MBF between mild disease (<50%) and moderate disease (50–70%). Also, patients with cardiomyopathy were excluded from our study. These patients commonly have reduced myocardial perfusion^30^, ^31^ and so the results presented here may not be applicable to patients with cardiomyopathy.

In summary, cardiac MRI with automated inline perfusion mapping is easy to implement and accurate for the detection of epicardial occlusive CAD with high sensitivity, strong negative predictive values, and adequate specificity. Practically, normal perfusion maps (all pixels encoding an MBF above the aforementioned cutoff values) are instantly recognizable and can rule out occlusive CAD, making this an ideal test to integrate into the clinical workflow.

## Conflict of Interest

No relationships with industry to declare.
